# Implementation of a Robotic Colorectal Surgery Program: Early Outcomes from a Tertiary Referral Center

**DOI:** 10.3390/jcm15208097

**Published:** 2026-10-19

**Authors:** Gaia Cicioni, Immacolata Iannone, Daniele Crocetti, Michelangelo Miccini, Daniele Biacchi, Enrico Spalice, Antonietta Lamazza, Michele Marchese, Enrico Fiori, Paolo Sapienza

**Affiliations:** 1 Department of Surgery, Sapienza University, 00161 Rome, Italy; gaia.cicioni@uniroma1.it (G.C.); immacolata.iannone@uniroma1.it (I.I.); daniele.crocetti@uniroma1.it (D.C.); michelangelo.miccini@uniroma1.it (M.M.); daniele.biacchi@uniroma1.it (D.B.); enrico.spalice@uniroma1.it (E.S.); enrico.fiori@uniroma1.it (E.F.); 2 Interdepartmental Unit of Digestive and Operative Endoscopy (UOSD), Department of Surgery, Sapienza University, 00161 Rome, Italy; antonietta.lamazza@uniroma1.it (A.L.); m.marchese@policlinicoumberto1.it (M.M.)

**Keywords:** robotic colorectal surgery, learning curve, perioperative outcomes, colorectal cancer, tertiary referral center

## Abstract

**Background:** Robotic-assisted colorectal surgery is increasingly adopted; however, evidence regarding outcomes during the early implementation phase remains limited. This study aimed to evaluate the feasibility and short-term outcomes of a newly established robotic colorectal surgery program at a tertiary referral center. **Methods:** All consecutive patients undergoing robotic colorectal resection between November 2019 and February 2026 were prospectively recorded and retrospectively analyzed. Perioperative outcomes, conversion rates, postoperative complications, in-hospital mortality, and histopathological results were assessed. **Results:** A total of 177 patients were included, with a progressive increase in surgical volume over time. Most procedures were performed for oncological indications (159, 90%), while 18 (10%) were performed for benign diseases. The most frequent procedures were right hemicolectomy (45%), left hemicolectomy (32%), and low anterior rectal resection (23%). Mean operative time was 331.1 ± 75.0 min. Conversion to open surgery occurred in 15 patients (8.5%), mainly due to extensive intra-abdominal adhesions, particularly during the early phase of the program. Postoperative complications occurred in 20 patients (11%), while 3 patients (2%) died during hospitalization. Major complications (Clavien–Dindo ≥ III) occurred in 7%, and anastomotic leakage in 3%. Mean hospital stay was 10.1 ± 7.6 days. R0 resection was reported in all cases, with adequate lymph-node retrieval for staging. **Conclusions:** In this single-center, non-comparative series, robotic colorectal surgery was feasible during program implementation, with acceptable short-term perioperative outcomes. Comparative studies are needed to determine its relative performance compared with laparoscopy.

## 1. Introduction

Minimally invasive surgery has become increasingly established as the preferred approach for the treatment of benign and malignant colorectal diseases, owing to its well-documented advantages in terms of faster postoperative recovery, shorter hospital stays, and reduced perioperative morbidity, without compromising oncological outcomes [1,2]. These benefits are largely attributable to decreased abdominal wall trauma, limited tissue manipulation, and improved visualization of deep anatomical structures, which are particularly relevant in pelvic surgery. Despite being a well-established technique, conventional laparoscopy has inherent technical limitations, especially in complex cases, including suboptimal instrument ergonomics, dependence on an assistant for camera control, two-dimensional visualization of the operative field, and restricted degrees of freedom. Such factors may translate into technical challenges and higher rates of conversion to open surgery. The introduction of robotic platforms was intended to overcome many of these constraints by providing high-definition three-dimensional vision, stable camera control, and articulated instruments with multiple degrees of freedom, allowing for precise dissection even within confined anatomical spaces such as the pelvis [3]. Consequently, robotic colorectal surgery has gained increasing adoption worldwide. Available evidence suggests that robotic surgery achieves postoperative and oncological outcomes comparable to those of laparoscopy, with some reports indicating lower conversion rates in high-volume centers [4]. However, most published data originate from institutions with long-established robotic programs, while information regarding outcomes during the early phases of implementation remains limited [5]. Since November 2019, our center has undertaken a structured program of robotic colorectal surgery with the aim of progressively integrating this technology into routine clinical practice. The present study evaluated the implementation of this program over its initial six-year period, focusing on perioperative safety, conversion rates, pathological outcomes, and temporal changes in surgical performance.

## 2. Patients and Methods

All operations were performed at Policlinico Umberto I (General Surgery and Minimally Invasive Unit, Department of Surgery, Sapienza University of Rome), a tertiary care university hospital. All consecutive patients who underwent colorectal robotic surgery at our institution between 1 November 2019 and 28 February 2026 were included in the present analysis. The decision to perform a robotic procedure was made within the multidisciplinary colorectal board and was not determined by randomization. Patient selection reflected the availability of the robotic platform, the acquired surgeon expertise, the anticipated technical complexity of the procedure (previous abdominal open surgery), and the feasibility of a minimally invasive approach. Institutional Review Board (IRB) approval was obtained (#2019/07/B–5, April 2019). Data were extracted in April 2026, after completion of the 30-day postoperative period for all patients. Patients operated on in 2026 (*n* = 7) were included in the overall analysis but excluded from the evaluation of annual volume trends because of incomplete-year data. All operations were performed using a da Vinci X surgical system (Intuitive Surgical, Sunnyvale, CA, USA) with a single-console configuration.

Eligibility criteria comprised age ≥ 18 years and provision of written informed consent for robotic-assisted surgery, including authorization for conversion to open surgery if required for intraoperative safety. Furthermore, all patients signed a specific authorization for the use of clinical data for research (AOU Policlinico Umberto I #0038763).

The indication for surgery was established by a multidisciplinary tumor board comprising colorectal surgeons, gastroenterologists, oncologists, radiologists, and pathologists. All patients underwent elective surgery following a standardized preoperative assessment and optimization pathway. Pre-admission evaluation included comprehensive laboratory testing, electrocardiography, formal cardiology clearance, and anesthesiology assessment. All patients underwent contrast-enhanced total-body computed tomography (CT) for staging purposes; in cases of rectal cancer, pelvic magnetic resonance imaging (MRI) was additionally performed to assess local tumor extension and mesorectal involvement. Endoscopic evaluation with colonoscopy was mandatory in all cases and included biopsy of the primary lesion for histological confirmation. When indicated, endoscopic tattooing was performed to facilitate intraoperative localization of the tumor. Mechanical bowel preparation and perioperative antibiotic prophylaxis were administered according to institutional protocols. All surgical interventions were performed by two surgeons (EF and PS), who operated sequentially during the study period and surgeon-specific case numbers and prior robotic experience were not prospectively recorded in a standardized manner, a surgeon-level learning-curve analysis was not performed. All procedures were performed under general anesthesia with orotracheal intubation, and patients received prophylactic antibiotics (cefazolin 2 g i.v.) at induction. Since 2025, all anastomoses have been routinely assessed for perfusion before and after their creation using indocyanine green near-infrared fluorescence. No drains were used for right hemicolectomy, whereas a single drain was used for low anterior resection and was routinely removed on the fourth postoperative day. The nasogastric tube was removed after surgery. The enhanced recovery after surgery (ERAS) protocol was adopted in 2025; therefore, patients received limited intravenous fluid administration in the ICU, and prophylactic antithrombotic therapy (heparin sodium 4000 U.I. s.c.) was discontinued once patients were fully mobilized. Follow-up consisted of clinical assessment and laboratory testing on postoperative day 2 and at 1 month, followed by outpatient visits every 6 months.

### 2.1. Study Population, Perioperative and Postoperative Variables

Demographic and clinical data were prospectively recorded and included age, sex, surgical indication, and preoperative physical status according to the American Society of Anesthesiologists (ASA) classification. Intraoperative parameters encompassed operative duration, defined as the interval from skin incision to final wound closure, and conversion to laparotomy. Postoperative outcomes included length of hospital stay, calculated as the number of postoperative nights spent in hospital, postoperative complications, and histopathological results. Recorded complications comprised anastomotic leak, intra-abdominal abscess, surgical site infection, pneumonia, Clostridioides difficile infection, and urinary tract infection. Histopathological assessment included resection margin status and lymph-node harvest, expressed as the total number of retrieved lymph nodes.

BMI, previous abdominal surgery, detailed comorbidity profiles, rectal tumor height, and neoadjuvant treatment were not systematically recorded in the study database and therefore could not be included in the present analysis.

### 2.2. Surgical Technique

In all cases, a standardized setup using three of the four available robotic trocars and two laparoscopic accessory ports was adopted, the latter serving for the camera and bedside assistance. Port placement was tailored according to the side of resection and the specific procedure performed, following a linear abdominal configuration to optimize instrument triangulation, minimize external arm collisions, and ensure adequate exposure of the operative field. Left-sided colonic and rectal resections were carried out using a medial-to-lateral approach, with early identification and division of the inferior mesenteric vessels when indicated and systematic visualization and preservation of the left ureter. The colon was mobilized along the white line of Toldt up to the splenic flexure, which was mobilized robotically at the surgeon’s discretion based on oncological and technical requirements. Rectal resections included both anterior and low anterior resections, with dissection performed above or below the peritoneal reflection and routine total mesorectal excision (TME) according to oncological principles. Right-sided colonic resections were likewise performed using a medial-to-lateral approach, with early identification and division of the ileocolic vessels and central vascular ligation in cases of malignancy. The right colon was fully mobilized along the white line of Toldt to the hepatic flexure. Anastomoses were fashioned using either intracorporeal (since 2025) or extracorporeal techniques. In extracorporeal cases, specimen extraction was performed through a protected extended port site using a wound protector. Left-sided and rectal anastomoses were typically created in an end-to-end fashion with a circular stapling device, whereas right-sided anastomoses were constructed in a side-to-side functional end-to-end configuration using a linear stapler. In patients with prior pelvic radiotherapy or in selected cases of complicated diverticular disease, a diverting loop ileostomy was performed. Throughout the procedure, advanced energy devices, Harmonic® scalpel (Ethicon, Cincinnati, OH, USA), were employed for dissection, and Hem-o-lok^®^ polymer clips (Teleflex, Morrisville, NC, USA) were systematically used for secure vascular control.

### 2.3. Data Extraction

Data were collected using Microsoft Excel 2024 (ver. 16.89.1) (Microsoft, Redmond, CA, USA) running on an iMac (Apple Inc., Cupertino, CA, USA) with macOS Monterey (ver. 12.7.6). Data were prospectively collected in an electronic anonymized database containing demographic characteristics, indication for surgery, procedural details, and postoperative complications. Final histopathology information was also obtained. The study was conducted according to the principles of the Declaration of Helsinki and approved by the Department of Surgery Research Committee. After completion of data extraction, a comparison of patients’ demographics, diagnosis, final histology, surgical technique, and postoperative course was performed.

### 2.4. Chronological Analysis

To explore the institutional learning curve, the cohort was divided into three consecutive phases: phase 1 (November 2019–December 2021, *n* = 19), phase 2 (January 2022–December 2023, *n* = 62), and phase 3 (January 2024–February 2026, *n* = 96). Operative time, conversion rate, overall and major (Clavien–Dindo ≥ III) complications, anastomotic leak, and length of stay were compared across phases. A cumulative sum (CUSUM) analysis of operative time and conversion was also performed. Outcomes before and after 2025 were compared in an exploratory analysis.

### 2.5. Statistical Analysis

Categorical variables are reported as absolute numbers and percentages, and continuous variables as mean ± standard deviation or median (interquartile range), according to their distribution as assessed with the Shapiro–Wilk test. Comparisons across chronological phases were performed with one-way ANOVA or the Kruskal–Wallis test for continuous variables, and with the chi-square or Fisher’s exact test for categorical variables; trends across phases were assessed with the Cochran–Armitage test. Outcomes before and after 2025 (introduction of ERAS, indocyanine green perfusion assessment, and intracorporeal anastomosis) were compared using the Mann–Whitney U test or chi-square/Fisher’s exact test. Missing data were not imputed, and denominators are reported for each variable. A two-sided *p* < 0.05 was considered statistically significant. Analyses were performed with SPSS 28.0.1.1 (IBM Corp., Armonk, NY, USA).

## 3. Results

The cohort included 177 patients who underwent robotic colorectal resections during the study period, with a progressive increase in annual surgical volume observed over time. After a relatively stable phase between 2019 and 2021 (5, 8, and 6 procedures, respectively), a steady increase was observed starting in 2022 (*n* = 27), followed by a steady increase in 2023 (*n* = 35), 2024 (*n* = 43), and 2025 (*n* = 46). Data for 2026 are partial (*n* = 7) and were not considered for trend evaluation.

Male patients accounted for 114 (64%), while 63 (36%) were female. The mean age was 69.0 ± 11.9 years (range, 38–90 years). A total of 73 (41%) patients were classified as American Society of Anesthesiologists (ASA) grade II, while 104 (59%) were classified as ASA grade III.

Most procedures were performed for oncological indications (159 [90%]), while 18 (10%) patients underwent surgery for benign disease, mainly due to recurrent diverticulitis or diverticular stenosis.

The distribution of procedures showed a predominance of right hemicolectomy, which was the most frequently performed operation (80 [45%]), followed by left hemicolectomy (56 [32%]) and anterior or low anterior rectal resection (41 [23%]). Additional procedures were performed in selected cases and included 11 (6%) protective ileostomies, 2 (1%) partial cystectomies for a sigmoid–vesical fistula, 5 (3%) cholecystectomies performed during right hemicolectomy, 1 (1%) cholecystectomy during left hemicolectomy, and 4 (2%) oophorectomies. The mean operative time was 331.1 ± 75.0 min (range, 170–540 min). Conversion to open surgery occurred in 15 (8.5%) patients.

The primary indication for conversion was extensive intra-abdominal adhesions (8 cases). Additional reasons included severe obesity (2 cases), lower rectal infiltration (2 cases), bleeding due to splenic injury (1 case), severe abdominal distension (1 case), and intolerance to pneumoperitoneum (1 case). All conversions occurred during the early years of the program.

Intracorporeal anastomosis was introduced in 2025 and subsequently performed in 22 patients (12.4% of the overall cohort), all of whom were included in phase 3. Accordingly, these procedures accounted for 22 of the 96 patients (22.9%) treated during phase 3.

Postoperative complications occurred in 20 (11%) patients; in-hospital mortality was 2% (3 patients).

According to the Clavien–Dindo classification (Table 1), 8 (4%) patients experienced minor complications (grade I–II), while 12 (7%) patients developed major complications (Clavien–Dindo ≥ III). Among these, 11 (6%) patients had complications of grade ≥ IIIB.

Surgical site infection and postoperative ileus were each observed in 1 patient (1%). Minor complications included one case of infective endocarditis managed with targeted antibiotic therapy, one case of postoperative fever of unknown origin, and one pleural effusion treated conservatively.

Anastomotic leakage occurred in 5 (3%) patients. Management strategies varied according to clinical presentation: one patient was treated conservatively with antibiotics (in the presence of a diverting ileostomy), one underwent percutaneous drainage combined with endoscopic stent placement (also with a protective ileostomy), and three required surgical re-intervention.

A Clavien–Dindo grade IVA complication was observed in one patient who developed a postoperative non-ST-elevation myocardial infarction requiring coronary angiography followed by cardiac surgery.

Regarding in-hospital mortality, all three patients were ASA III, aged 74, 78, and 89 years, and had undergone elective surgery for oncological disease. One patient experienced intraoperative cardiac arrest. Two additional deaths were due to septic shock: one secondary to KPC-related pneumonia and one due to polymicrobial infection (KPC and *Escherichia coli*) without anastomotic leak. 

The mean hospital stay after surgery was 10.1 ± 7.6 days (range, 3–51 days).

Of the 177 patients included in the study, 159 underwent surgery for a suspected or confirmed neoplastic lesion, while 18 were operated on for diverticular disease. Among the 159 patients operated on for a suspected or confirmed neoplastic lesion, pathological TNM staging was available in 131 (82%): stage 0–I in 39 (30%), stage II–III in 88 (67%), and stage IV in 4 (3%). In the remaining 28 patients, complete TNM staging was not available. This group included patients with incomplete pathological or staging information and patients who had undergone surgery for lesions considered suspicious for malignancy based on preoperative imaging and/or biopsy findings, but whose final histopathological examination demonstrated a completely benign lesion and therefore did not allow oncological TNM classification.

An R0 resection was achieved in all 159 patients who underwent surgery for a suspected or confirmed neoplastic lesion, according to the pathological assessment of the resection margins. Lymph-node metastases were identified in 41 of 159 patients undergoing surgery for suspected or confirmed neoplastic disease (26%).

**Figure 1:**
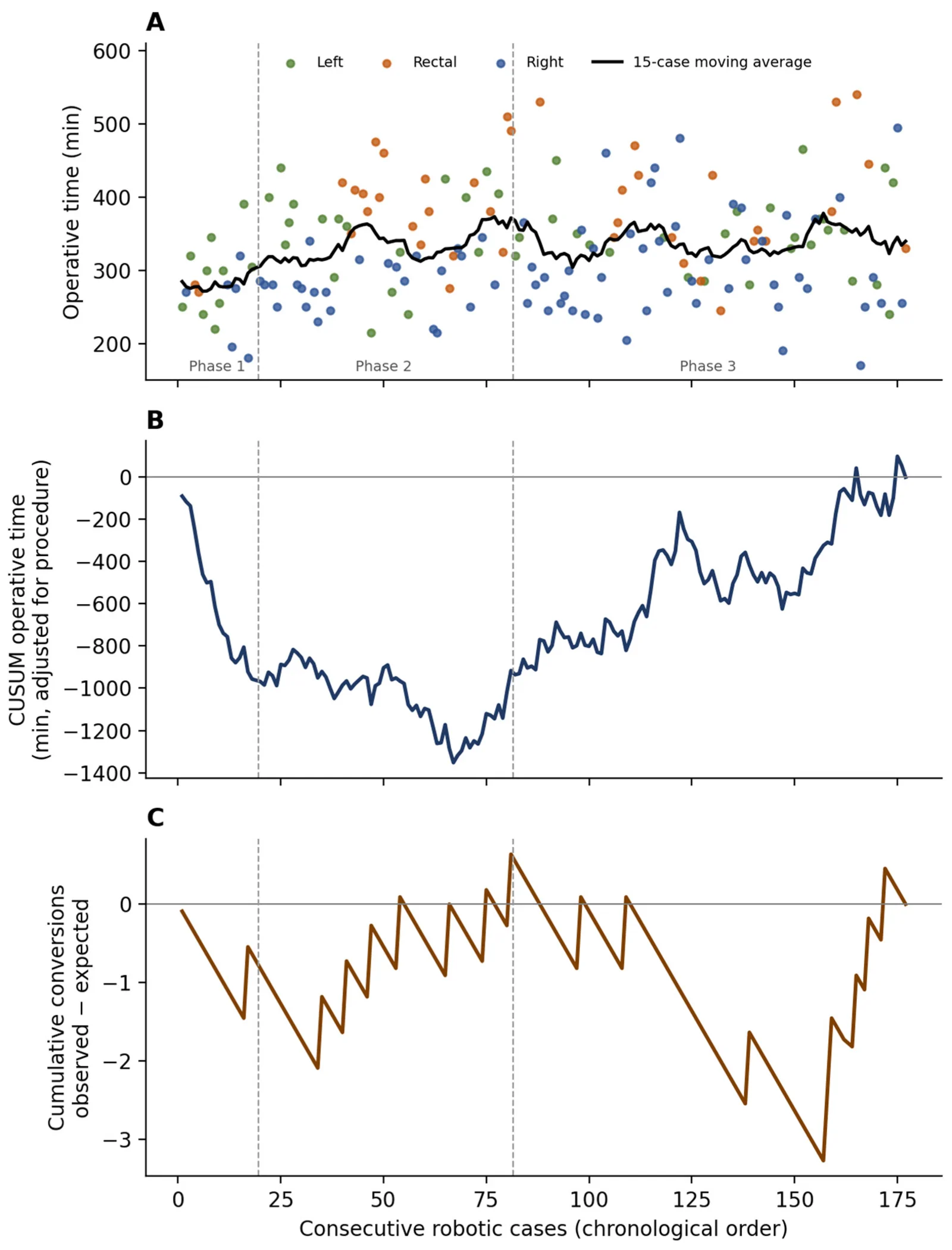
Operative time and conversion over the course of the program. (**A**) 15-case moving average. (**B**) CUSUM of operative time, calculated as the cumulative deviation of each case from the mean of its procedure type; a rising curve indicates operative times longer than expected. (**C**) Cumulative sum of observed minus expected conversions (expected = overall rate, 9.1%). Dashed lines separate phases 1, 2, and 3.

To explore temporal changes potentially reflecting the learning process during implementation of the robotic colorectal surgery program, the cohort was divided into three consecutive chronological phases, and the main perioperative outcomes were compared across these periods (Table 2). Mean operative time was 277.5 ± 51.5, 337.6 ± 72.2, and 337.1 ± 77.1 min in phases 1, 2, and 3, respectively (*p* = 0.005). Conversion to open surgery occurred in 1/19, 7/62, and 8/96 patients (*p* = 0.74). Major complications (Clavien–Dindo ≥ III) occurred in 3, 4, and 5 patients (*p* = 0.22), and median length of stay was 10, 8, and 7 days (*p* < 0.001) (Table 1). The CUSUM analysis of operative time showed no learning-curve pattern of decreasing operative time: the curve declined until approximately case 67 and then progressively rose (Figure 1). Furthermore, we noted a shorter length of stay (7 vs. 8 days, *p* < 0.001) when comparing patients operated on starting from 2025 (*n* = 53) with those operated on between 2019 and 2024 (*n* = 124). No significant differences were observed in overall complications (6 [11%] vs. 15 [12%], *p* = 1.00) and anastomotic leak (3 [2%] vs. 2 [1%], *p* = 0.16).

## 4. Discussion

The present study reports the initial experience of a robotic colorectal surgery program implemented in a tertiary referral center. Our findings indicate that the introduction of robotic technology into routine colorectal practice was feasible, with perioperative morbidity, conversion rates, and pathological outcomes documented throughout the study period. Importantly, these results were obtained in a real-world setting, outside high-volume centers with long-standing robotic expertise, thereby reflecting some of the practical challenges of program implementation.

Minimally invasive colorectal surgery has evolved significantly over recent decades, with laparoscopy representing the standard approach in many institutions [6]. However, well-recognized technical limitations—particularly in confined anatomical spaces such as the pelvis—have driven the increasing adoption of robotic platforms. In this context, the enhanced three-dimensional visualization and improved instrument dexterity offered by robotic systems may facilitate more precise dissection and improve surgeon ergonomics, particularly during complex procedures.

Conversion rate represents a relevant quality indicator during the implementation of a robotic colorectal program [7,8,9]. In our cohort, conversion to open surgery occurred in 8.5% of cases. As our study lacks a comparison group, this finding is descriptive and does not indicate an advantage of the robotic approach. The main cause of conversion was extensive intra-abdominal adhesions, suggesting that patient and disease-related factors contributed substantially to conversion in our series.

A relevant finding was the progressive adoption of intracorporeal anastomosis. This shift may reflect increasing familiarity with and technical experience of the robotic platform [10]. Intracorporeal reconstruction may offer advantages such as reduced bowel manipulation and improved precision, facilitated by enhanced visualization and instrument dexterity [11]. In our series, its implementation was not associated with an apparent adverse effect on perioperative outcomes, although the non-comparative design and limited number of cases prevent definitive conclusions regarding its safety or benefits during program implementation. In addition, the temporal distribution of operative time and conversion rates should be interpreted cautiously, as the CUSUM analysis did not demonstrate a conventional pattern of progressive improvement in operative time. Postoperative morbidity in our study was consistent with the available literature [12]. Overall complication rates remained within acceptable limits, and the incidence of major complications (Clavien–Dindo ≥ III) was comparable to that reported in similar minimally invasive series. The anastomotic leak rate of 3% further supports the technical reliability of robotic colorectal surgery. Three (2%) patients died during hospitalization, a figure that is not negligible and deserves consideration. Two deaths were due to septic shock from multidrug-resistant nosocomial infections and one to an intraoperative cardiac arrest, in patients with a high comorbidity burden. The three deaths occurred in the initial phase of the program, and the small number of events does not allow any inference on a relationship with the learning curve. In-hospital mortality should be interpreted considering the age (mean 69 years) and ASA status (59% ASA III) of our population and the local epidemiology of carbapenem-resistant infections.

The implementation of the robotic approach for colorectal cancer at our Institution was compatible with the maintenance of oncological resection principles [13]. Furthermore, the heterogeneous distribution of tumor stages indicates that the robotic approach was applied across a broad spectrum of disease rather than being restricted to highly selected early-stage tumors. This aspect provides information on the applicability of the program in routine clinical practice, although the absence of long-term oncological follow-up limits interpretation of these findings.

This study has several limitations. First, it represents a single-center experience, which may limit generalizability. Second, the absence of a direct laparoscopic control group prevents definitive conclusions regarding superiority or comparative advantages of the robotic approach [14]. Third, although data were prospectively collected, the analysis remains retrospective. Fourth, ERAS, indocyanine green perfusion assessment, and intracorporeal anastomosis were all introduced in 2025; outcomes before and after this date may not be directly comparable, and the effect of each individual change cannot be isolated. Fifth, two surgeons operated sequentially, and case mix changed over time, so the learning-curve analysis reflects the experience of the program as a whole and cannot be attributed to increasing proficiency alone; phase 1 included only 19 patients. Sixth, BMI, previous abdominal surgery, detailed comorbidity profiles, rectal tumor height, and neoadjuvant treatment were lacking in 41 cases, thus limiting our statistical analyses. Seventh, tumor staging was unavailable in 28 patients. Finally, long-term oncological outcomes are not yet available and will require further follow-up.

We believe that as surgical proficiency increases and familiarity with the robotic platform improves, the perceived advantages in terms of visualization, precision, and ergonomics may become progressively more evident. In this context, reverting to conventional laparoscopy after gaining extensive experience with robotic surgery may become increasingly challenging, not only from a technical standpoint but also in terms of surgical workflow and operator comfort. This phenomenon may reflect a broader technological transition rather than a simple change in surgical technique. However, whether robotic surgery should become the preferred approach for complex colorectal procedures cannot be determined from the present non-comparative series.

## Figures and Tables

**Table 1:** Postoperative complications by Clavien–Dindo grade (*n* = 177).

Grade	*n* (%)	Events
I–II	8 (4%)	Endocarditis, fever of unknown origin, pleural effusion, subclinical anastomotic fistula, intestinal obstruction
IIIa	1 (1%)	anastomotic fistula
IIIb	7 (3%)	Recto-vaginal fistula, anastomotic dehiscence, rectal perforation, anastomotic dehiscence, necrosis of the colostomy, anastomotic dehiscence, and adhesive band obstruction
IVa	1 (1%)	NSTEMI requiring coronary angiography and cardiac surgery
V	3 (2%)	Intraoperative cardiac arrest; septic shock (KPC pneumonia); septic shock (polymicrobial, KPC + *E. coli*)
Total	20 (11%)	Major (≥III): 12 (7%). Anastomotic leak: 5 (3%). SSI: 1. Ileus: 1

**Table 2:** Outcomes by chronological phase.

Variable	Phase 1 2019–21 (*n* = 19)	Phase 2 2022–23 (*n* = 62)	Phase 3 2024–26 (*n* = 96)	*p*	Test
Age, years, mean ± SD	69.8 ± 12.8	68.9 ± 11.5	68.8 ± 12.0	0.95	ANOVA
ASA III, *n/N* (%)	9/10 (90)	34/62 (55)	54/95 (57)	0.10	Fisher–Freeman–Halton
Rectal resections, *n* (%)	2 (10)	19 (31)	20 (21)	0.18	Fisher–Freeman–Halton
Operative time, min, mean ± SD (*n*)	277.5 ± 51.5 (18)	337.6 ± 72.2 (62)	337.1 ± 77.1 (94)	0.005	ANOVA
Operative time adjusted for procedure, min	276	332	341	–	OLS; vs. phase 1: *p* = 0.002 and <0.001
Conversion to open surgery, *n/N* (%)	1/19 (5)	7/62 (11)	8/96 (8)	0.74	Fisher–Freeman–Halton
Any complication, *n* (%)	8 (42)	5 (8)	7 (7)	<0.001	Fisher–Freeman–Halton
Major complications (CD ≥ III), *n* (%)	3 (16)	4 (6)	5 (5)	0.22	Fisher–Freeman–Halton
Anastomotic leak, *n* (%)	0	2 (3)	3 (3)	1.00	Fisher–Freeman–Halton
In-hospital mortality, *n* (%)	2 (10)	1 (2)	0	0.013	Fisher–Freeman–Halton
Length of stay, days, median (IQR)	10 (9–17)	8 (8–9)	7 (7–8)	<0.001	Kruskal–Wallis
Lymph nodes retrieved, mean ± SD (*n*)	15.0 ± 6.3 (16)	17.8 ± 6.9 (49)	17.2 ± 8.0 (82)	0.30	Kruskal–Wallis
Intracorporeal anastomosis, *n* (%)	0	0	22 (23)	<0.001	Fisher–Freeman–Halton

CD, Clavien–Dindo; FFH, Fisher–Freeman–Halton exact test; OLS, ordinary least squares including phase and procedure type. Phase 3 includes 7 patients operated on in 2026. Percentages in the ASA row refer to patients with available ASA class (missing in 10). Conversion data are missing in 1 patient (phase 3). Anastomotic leak = dehiscence/fistula of the anastomosis. Length of stay = postoperative day of discharge.

## Data Availability

The data supporting the findings of this study are available from the corresponding author upon reasonable request, subject to institutional approval and applicable data protection regulations. The data are not publicly available due to privacy and ethical restrictions concerning patient data.
